# Computational Inference of Synaptic Polarities in Neuronal Networks

**DOI:** 10.1002/advs.202104906

**Published:** 2022-03-31

**Authors:** Michael R. Harris, Thomas P. Wytock, István A. Kovács

**Affiliations:** ^1^ Department of Physics and Astronomy Northwestern University Evanston IL 60208 USA; ^2^ Department of Physics Loyola University Chicago Chicago IL 60660 USA; ^3^ Northwestern Institute on Complex Systems Northwestern University Evanston IL 60208 USA

**Keywords:** *Caenorhabditis elegans*, complex networks, connectome, link prediction, synaptic polarity

## Abstract

Synaptic polarity, that is, whether synapses are inhibitory (−) or excitatory (+), is challenging to map, despite being a key to understand brain function. Here, synaptic polarity is inferred computationally considering three experimental scenarios, depending on the nature of available input data, using the *Caenorhabditis elegans* connectome as an example. First, the inputs consist of detailed neurotransmitter (NT) and receptor (R) gene expression, integrated through the connectome model (CM). The CM formulates the problem through a wiring rule network that summarizes how NT‐R pairs govern synaptic polarity, and resolves 356 synaptic polarities in addition to the 1752 known polarities. Second, known synaptic polarities are considered as an input, in addition to the NT and R gene expression data, but without wiring rules. These data train the spatial connectome model, which infers the polarity of 81% of the CM‐resolved connections at >95% precision, while also inferring 147 of the remaining unknown polarities. Last, without known expression or wiring rules, polarities are inferred through a network sign prediction problem. As an illustration of high performance in this case, the generalized CM is introduced. These results address imminent challenges in unveiling large‐scale synaptic polarities, an essential step toward more realistic brain models.

## Graphical abstract

In this paper, a sequence of computational methods to infer missing synaptic polarity information (excitatory ‐ blue, inhibitory ‐ red), both with and without input on neurotransmitter and receptor gene expression as well as on neurotransmitter‐receptor wiring rules is introduced.

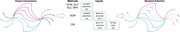

## 1 Introduction

The vast scale and unprecedented complexity of the human neuronal network (i.e., the “connectome”) poses an inherent challenge to achieving large‐scale maps of both the connectivity of neurons and their excitatory or inhibitory nature, the latter referred to as synaptic polarity. Synaptic polarity is essential to understand how neural circuits work: we must know whether the synapses are excitatory (positive) — increasing the likelihood that the postsynaptic partners fire — or inhibitory (negative), muffling the partners instead. While methods to map connectivity are rapidly increasing their capacity for high‐throughput identification of synapses between neurons, in both humans and model organisms,^[^ ^1^, ^2^, ^3^ ^]^ methods to observe the polarity dynamically remain low‐throughput and sporadic,^[^ ^4^, ^5^ ^]^ calling for computational methods that can infer synaptic polarity from limited information.^[^ ^6^ ^]^

A natural model organism to explore such computational methods is the roundworm *Caenorhabditis elegans*, owing to its well‐characterized genetic determinants of brain development, and synaptic connectome (http://wormwiring.org).^[^ ^7^, ^8^, ^9^ ^]^ Yet, even in this model organism, the polarity of synapses is largely untested due to experimental difficulties in determining whether a connection is inhibitory or excitatory.^[^ ^5^, ^10^, ^11^, ^12^, ^13^, ^14^ ^]^ Dynamical simulation efforts, such as Sim‐CE^[^ ^15^ ^]^ and OpenWorm,^[^ ^16^ ^]^ would benefit from integrating reliable synaptic polarity as an input in an effort to better model organism behavior.

**Figure 1:**
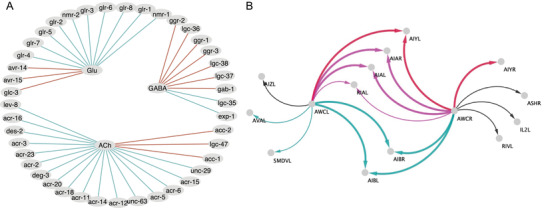
Illustration of the *Caenorhabditis elegans* input datasets: A) Network of synaptic wiring rules responsible for the polarity of the connections between three neurotransmitters and 42 receptors expressed by the pre‐ and postsynaptic neurons, respectively. Excitatory rules are shown in blue, while inhibitory rules in red. Each neurotransmitter has multiple known rules of unconventional polarity.^[^ ^6^ ^]^ B) Postsynaptic partners of the glutamergic AWC neurons, highlighting a balanced amount of excitatory (blue) and inhibitory (red) connections, according to Fenyves et al.^[^ ^6^ ^]^ Only connections where the postsynaptic partner has at least one receptor expressed from panel (A) are shown. Thicker lines indicate experimental polarity information.^[^ ^10^, ^11^ ^]^ The AWC–AIA complex connections are only partially confirmed, as they are expected to be inhibitory.^[^ ^11^ ^]^ In this paper, we aim to computationally resolve the complex (magenta) and unknown (black) polarities at the connectome level.

Recently, the neurotransmitter‐receptor (NT‐R) wiring rules have been curated in *C. elegans* from decades of literature studies to assign synaptic polarities between 295 neurons (out of a total of 302) using both NT and R expression.^[^ ^6^ ^]^ We gathered the known connectome of chemical synapses, the sign polarity rules between three main neurotransmitters (NT) and 62 receptors (R), and the expression of the associated genes from ref. [6]. Currently, out of 62 Rs, only 42 are found to be involved in at least one wiring rule and expressed in at least one of the neurons. As indicated in **Figure** **1**, our current understanding of the governing rules between these three NTs and 42 Rs is much more complex than the traditional paradigm of inhibitory GABA‐ergic synapses and excitatory cholinergic or glutamergic synapses. Clearly, unconventional postsynaptic effects of NTs prevail, such as cholinergic^[^ ^17^, ^18^ ^]^ and glutamatergic inhibition,^[^ ^10^, ^11^, ^19^, ^20^ ^]^ meaning that a neuron can excite some while inhibiting some other postsynaptic partners using the same NT, as illustrated in Figure 1B. Such synaptic complexity has been identified as a mechanism behind learning and synaptic plasticity.^[^ ^21^ ^]^

**Figure 2:**
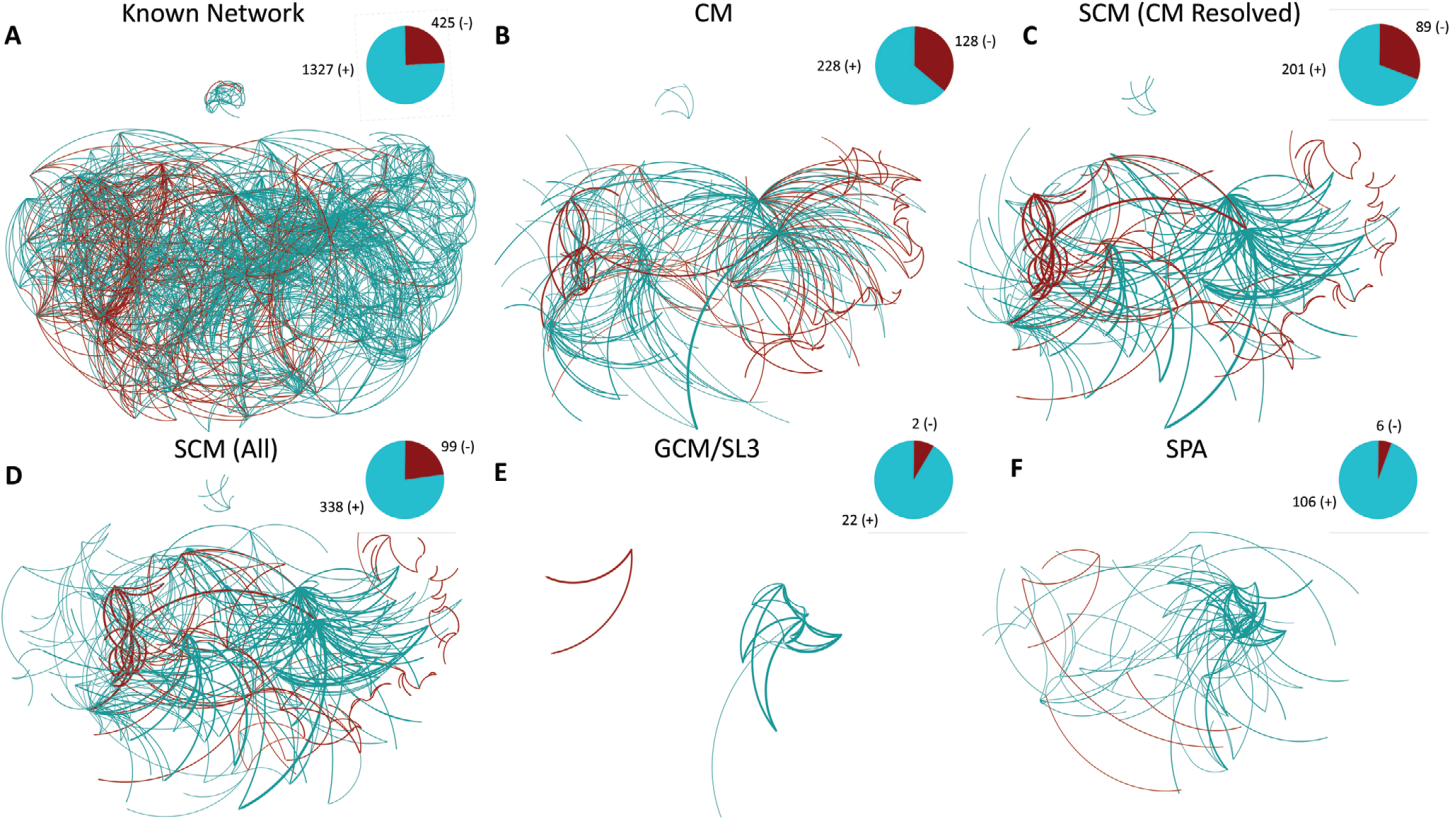
Known and inferred polarities: A) Network visualization of 1752 synaptic connections of known polarity from ref. [6]. In this and subsequent panels, the number of inhibitory (−) and excitatory (+) connections is indicated by a pie chart. B) The network of 356 connections characterized as “complex” by ref. [6], and resolved to either positive or negative polarity via the CM. C) The subset of connections in (B), resolved by the SCM at 95% precision. D) All inferred synaptic polarities by the SCM at a threshold corresponding to 95% precision. E,F) Network of novel polarity predictions made with SL3 (E) and SPA (F) at 95% precision. In panels (B–F), thicker lines indicate top predictions with larger absolute prediction scores.

As a conclusion of ref. [6], the polarity of each input–output neuron pair (or “connection”) with presynaptic NT and postsynaptic R expression was assigned into one of the following four categories: known (425 negatives, 1327 positives, **Figure** **2**A) or unresolved (471 complex, 623 unknown) polarities. Note that in addition to these 623 unknown connections, there are 792 connections left out of our analyses, as they lack either presynaptic NT or postsynaptic R expression. Also, the actual number of synapses is an order of magnitude higher, as the same connection can manifest in a number of individual synapses between two neurons. In lack of additional information on individual synapses, we address synaptic polarity at the level of connections, instead of individual synapses, as ref. [22] has done at the subcircuit level.

In this paper, we systematically extend the recently introduced connectome model (CM)^[^ ^23^ ^]^ to significantly expand the network of known synaptic polarities. As a starting point, we show that the literature‐curated wiring rules^[^ ^6^ ^]^ are compatible with the CM, readily resolving most (76%) of the complex interactions, when both expression and wiring rule information is used. Then, we show that by taking the known polarities as an input, the CM can be extended to infer 20% of the remaining 738 unresolved polarities at an estimated 95% precision, even without using any wiring rules as an input.

In higher organisms, such as humans, currently we not only lack a comprehensive list of wiring rules, but we are also far from a sufficient dataset of spatially resolved, single‐neuron‐level NT and R expression profile. Is it possible to expand partially known synaptic polarities to the rest of the connectome even in lack of any genetic information? Addressing this question, last, we show that using only the signed network patterns of the connectome can yield top predictions in agreement with the CM.

## 2 Results

Here, we provide three extensions of the original CM to infer synaptic polarities, each corresponding to a different scenario depending on the nature of available input datasets, for illustration, see the Table of Contents figure. First, we consider the best case scenario of detailed knowledge on both gene expression and wiring rules. Next, we address the more common case when no reliable wiring rules are available but some synaptic polarities have been determined. Last, we consider the current situation of the human synaptic connectome, where a subnetwork of synaptic polarities are known, but without matching expression profiles. For this case, we also introduce a number of signed network‐based approaches.

### 2.1 Integrating Gene Expression and Wiring Rules: Connectome Model

We start with the observation that the inhibitory and excitatory synaptic wiring rules^[^ ^6^ ^]^ can be combined into a signed NT‐R wiring rule network (Figure 1A). In this signed wiring rule network (O), each NT (*i*) is connected to each R (*j*) with a positive (*O*_*ij*_ = 1) link if their matching expression results in excitatory synapses, *O*_*ij*_ = −1 if inhibitory, and *O*_*ij*_ = 0 otherwise. The concept of a wiring rule network has been the essence of the recently introduced CM,^[^ ^23^ ^]^ applied first to the electric (gap junction) connectome of the *C. elegans*. Motivated by that work, here, we assign weights to a signed, directed neuronal network as

(1) $$B={\mathrm{XOY}}^{⊺}$$

where B is determined from the expression of NT genes in the presynaptic neurons (X), the expression of R genes in the postsynaptic neurons (Y), and the wiring rule network O that specifies how NT‐R combinations contribute to sign polarities. In the CM, the matrix element *B*_*ij*_ represents the synaptic connection from neuron *i* to *j*. The interpretation of Equation (1) is that a positive rule (*O*_*kl*_ = 1) contributes to the polarity of a synapse from *i* to *j* if neuron *i* expresses NT *k* (*X*_*ik*_ > 0) and neuron *j* expresses R *l* (*Y*_*jl*_ > 0). The input datasets for X, Y, and O are known from a recent literature curation effort for most (295 out of 302) *C. elegans* neurons.^[^ ^6^ ^]^ In comparison, the original CM was designed for the special case of only one relevant expression profile, that is, when *Y* = *X*, resulting in undirected networks *B* = *B* ^*T*^. As *X* and *Y* can be different in Equation (1), we generally arrive at directed predictions, where *B* ≠ *B* ^*T*^.

In ref. [6], 1752 directed neuron pairs have been identified as either positive or negative, based on only consistent rules of the same sign, summarized in the network of known polarities (Figure 2A). This approach left 1094 connections unresolved, with those supported by both positive and negative NT‐R rules falling into the “complex” category, while the rest of the pairs fall into the “unknown” category.

In contrast, here we propose to identify synaptic polarities with the sign of the weights identified in Equation (1), A=sign(B), leading to the CM of synaptic polarities:

(2) $$A=\mathrm{sign}({\mathrm{XOY}}^{⊺})$$

When considering the elements of *A*_*ij*_ that are present in the synaptic connectome, Equation (2) not only provides exactly the inferred positive and negative synaptic polarities in ref. [6], but it also resolves most of the complex polarities (356 out of 471, Figure 2B), a 20% overall increase in resolved polarities. At this stage, application of the CM is rather transparent. For example, a complex polarity is resolved as a negative pair when a neuron pair has more negative rules than positives and vice versa. On the example of AWC neurons in Figure 1B, the CM resolves the AWCL – RIAL and AWCR – RIAL connections as positive. The reason is that these connections are supported by four positive NT‐R rules (Glu vs glr‐1, glr‐2, glr‐3, and glr‐6) with only 1 negative rule (Glu vs avr‐15). The rest of the 738 unresolved synaptic polarities cannot be inferred by being contingent on both the currently known genetic expression and wiring rules. However, as we show next, synaptic polarities can be inferred with high precision even in this yet unresolved space, if we consider some of the known synaptic polarities as an input.

### 2.2 Integrating Gene Expression and Known Synaptic Polarities: Spatial Connectome Model

We have shown that having the NT and R expression profiles as well as the NT‐R wiring rules at hand, the CM can resolve most complex connections. The key to extend synaptic polarities further is to obviate the dependency on known wiring rules. Without wiring rules we cannot assume full access to B either, as the weights are derived from the wiring rules. Therefore, we are restricted to use the known unweighted, signed network of synaptic polarities, A, as an input for the spatial connectome model (SCM). The same scenario arises naturally when synaptic polarities are known from independent experiments, such as patch clamping.^[^ ^4^ ^]^

**Figure 3:**
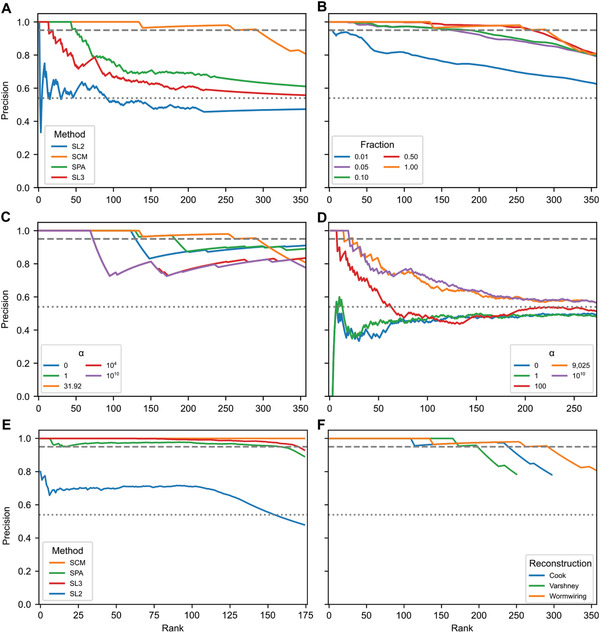
Computational validation: A) Comparison of the synaptic polarities inferred by our proposed methods versus the resolved polarity of complex connections by the CM, see text for details. The dotted line indicates the random expectation (0.54) with the same fraction of positives (64%) as in the CM‐resolved polarities. In panels (A)‐(D) and (F), the orange curve corresponds to the optimal α = 31.92 regularization value, while the dashed line indicates 95% precision. B) Precision of the optimally regularized SCM against the CM‐resolved connections, when using only a fraction of the known network as the input network. Notably, with only 5% of the connections available, approximately one‐half of the complex connections can still be resolved with 95% precision. C) Precision of the regularized SCM for various values of the α regularization hyperparameter indicated in the legend. D) Precision of the regularized GCM for various values of α, tested against the CM‐resolved connections. E) Tenfold cross validation of the SCM and three network‐based methods on the known network. In contrast to all other panels, the predictions are tested against the test fold, see Section 4. F) Precision of the optimally regularized SCM against the CM‐resolved connections, in comparison to that in two alternative connectome reconstructions.^[^ ^8^, ^9^ ^]^

**Figure 4:**
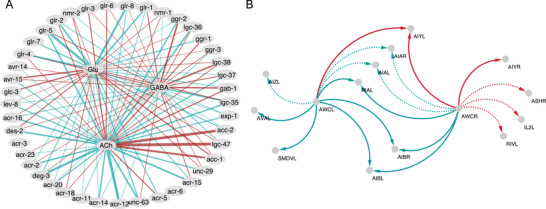
Predictions of the spatial connectome model: A) The ‘minimal’ rule network ($\tilde{O}$) of the SCM, an intermediate, formal step to extend known polarities to the rest of the connections, see Section 4. Thicker lines correspond to larger absolute values of the identified minimal rule weights. B) SCM‐resolved polarities of the AWC example in Figure 1B. Solid (dashed) lines indicate known polarities and those inferred above (below) the chosen 95% precision threshold.

Within the SCM, we first use the NT and R expression to reconstruct a formal, minimal set of wiring rules that optimally satisfy the SCM equation (see Section 4):

(3) $$A={\mathrm{XOY}}^{⊺}$$

where the element‐wise nonlinear sign function in Equation (2) is omitted for tractability. To find the corresponding optimal $\tilde{O}$ matrix, it is essential to disregard all neuron pairs without synaptic connection and even those without known synaptic polarity, as those pairs have no predictive information on the rules. To incorporate such (spatial) constraints to Equation (3), we follow the steps taken in ref. [23]. First, we need to reorganize A to a=vec(A), where vec is an operator that reshapes a matrix of *N* rows and *M* columns into a vector of *NM* elements row‐by‐row. Then, the resulting vector is truncated into $a^{\prime }$, retaining only entries of a that correspond to observed connections of known polarity. Reshaping the left‐hand side of Equation (3) induces a corresponding change in the right‐hand side, that is, K=X⊗Y and o=vec(O), where ⊗ is the Kronecker product. Then, $K^{\prime }$ represents the truncated version of this matrix that summarizes the expression information of only neuron pairs connected by a connection of known polarity. At this stage, the signs in the integrated network are encoded in $a^{\prime }$, and the expression information is encoded in $K^{\prime }$. These are inputs to the SCM equation

(4) $$a^{\prime }=K^{\prime }o$$

which can be solved for a minimal $\tilde{o}$ (in the Frobenius norm) by ridge regression techniques,^[^ ^23^, ^24^ ^]^ depending on an α regularization hyperparameter, see Section 4. Then, at the optimal α = 31.92 value, see **Figure** **3**C, the obtained minimal rule weights vector $\tilde{o}$ can be rearranged into a matrix format, $\tilde{O}$, illustrated as a wiring rule network in **Figure** **4**A.

Now, that we have a formal, minimal solution of the unknown rule network, we go beyond the SCM approach by accepting $\tilde{O}$ as an input for an updated CM. To be precise, $\tilde{O}$ is substituted into Equation (1) together with the original NT and R expression, to obtain polarity predictions for all connections. These prediction scores are then ranked based on the absolute value of the entries in descending order. We then tested the inferred synaptic rules against the complex pairs that have been resolved by the CM, serving as a ground truth data. As shown in Figure 3A, the SCM identified exactly (with 100% precision) the polarity of the top ranked 134 pairs, meaning that they are in complete agreement with the CM‐resolved polarities. At a more relaxed, yet still rather strict, requirement of >95% precision, the SCM inferred the top 290 connections, illustrated in Figure 2C. The SCM's performance is remarkable, considering that the wiring rules are not used as an input.

The SCM not only provides predictions for the 356 resolved complex polarities, but it also infers the sign of the remaining 738 unresolved pairs with NT‐R expression (115 unresolved complex pairs and 623 of the unknown pairs). At the same time, the SCM predictions are perfectly in line with the polarities in the known network. Within the subset of CM‐resolved complex connections >95% precision corresponds to an absolute prediction score value of ⩾0.2154. By considering only predictions above this score, we expect similarly high precision for the inferred 147 unresolved (out of 738) connections, even though we no longer have reliable ground truth data to validate these predictions. As an indication of high‐quality predictions, nine out of these 147 predictions are inferred with a score higher than the 0.59 value corresponding to perfect precision in the subset of complex pairs. Altogether, 40% (437 out of 1094) of the unresolved synaptic pairs have been resolved by the SCM alone, at an estimated precision of at least 95%, as illustrated in Figure 2D. Going back to the example of AWC neurons in Figure 1B, in addition to the CM‐resolved pairs, SCM also provides a sign for the rest of the connections, although those might be less reliable, as they fall below the 95% precision threshold, see Figure 4B.

At this point, the question arises of how complete the input network needs to be to enable reliable inference. Do we need to have experimental access to most polarities before we could computationally expand them to the rest of the network? To address this question, we considered using only a fraction of the known network as an input, validated against the 356 CM‐resolved complex polarities. As illustrated in Figure 3B, the SCM predictions are highly robust, even when only 5% of the known connections are used as an input.

### 2.3 Inferring Synaptic Polarities Based on Known Synaptic Polarities Only

Application of the SCM demonstrates the ability to infer polarities when the NT‐R expression of neurons is well known, but in practice synaptic connectivity is often collected without matching expression information. Furthermore, when proceeding from *C. elegans* to higher organisms, acquisition of synaptic connectivity and spatially resolved expression information is made more difficult by the lack of regularity in connectivity from individual to individual, as well as by the difficulty of spatially resolving the single cell expression data. Therefore, establishing methods that can infer polarities relying solely on signed network patterns of known polarities will prove valuable.

#### 2.3.1 Generalized Connectome Model

Here, we show that a generalized CM (GCM) can be applied even in the complete lack of genetic information. We start from Equation (3) and introduce the notation $U={\mathrm{OY}}^{⊺}$, yielding A=XU. This linear equation can be formally solved by ridge regression, leading to $X={\mathrm{AU}}^{+}$, where $U^{+}$ stands for the (optionally regularized) generalized inverse of U. Similarly, we can approximate $Y^{⊺}$ as $Y^{⊺}=V^{+}A$, with V=XO. Note that we do not apply any truncation during these solutions. With these inverse solutions, we arrive at the GCM equation

(5) A=AWA

with the notation of $W=U^{+}{\mathrm{OV}}^{+}$ standing for the unknown generalized rule matrix. The resulting GCM only uses the signed connectome as an input. The GCM can be solved analogously to the SCM, via vectorization and spatial truncation, resulting in a minimal $\tilde{W}$ rule matrix, leading to synaptic polarity predictions as $A^{*}=A\tilde{W}A$. Note that the condition to have a minimal $W=U^{+}{\mathrm{OV}}^{+}$ is generally different from the original formulation seeking a minimal O. Also note that in contrast to the SCM, the prediction of the GCM is nonlinear in the input network, A. One might think that due to this nonlinearity and the additional approximations made during the derivation of the GCM equation, it is harder to satisfy Equation (5). In fact, the situation is the opposite. Regularization and truncation is a key part of the GCM, as without those Equation (5) is automatically satisfied with $\tilde{W}=A^{†}$, the Moore–Penrose pseudoinverse of A. This unregularized limit is (over)fitting the data exactly without inferring any new polarities. As shown in Figure 3D, the heavily regularized limit is found to work best, when α → ∞. As we show next, this finding is related to recent advances in network‐based link prediction.

#### 2.3.2 Connection to Network‐Based Sign Prediction

Interestingly, the proposed GCM is not only a generalization of the SCM, but it can also be considered as a signed extension of the L3 method,^[^ ^25^ ^]^ a network‐based inference method utilizing paths of length three. Indeed, in the strongly regularized case of α → ∞ the solution of the GCM simplifies into $A^{+}\left(\alpha \right)\propto A^{⊺}$, readily providing the signed length 3 (SL3) formula $A^{\prime }\propto {\mathrm{AA}}^{⊺}A$ introduced in the Section 4, Equation (8). We have therefore established that the GCM is a joint generalization of the SCM and L3, encapsulating the finding that the connectivity patterns of the neurons (i.e., the A signed adjacency matrix) can be used as a proxy for the underlying genetic features governing neuronal network wiring.

**Figure 5:**
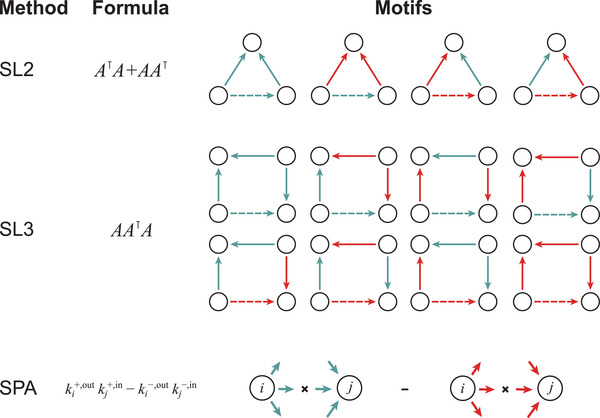
Summary of the signed extension of basic network‐based methods: The table summarizes each of signed length 2 (SL2), signed length 3 (SL3), and signed preferential attachment (SPA), their mathematical expression, and the contributing network patterns. Dashed edges indicate the predicted polarity. See text for further discussion of the formulas.

Yet, as the GCM uses much more limited information than the SCM, we cannot expect to unveil all missing synaptic polarities at a similar precision. Still, with appropriate thresholding, the same >95% (in fact 100%) precision can be maintained for the top 13 complex predictions of SL3, corresponding to an absolute prediction score of ⩾26. At the same threshold, SL3 unveils an additional 11 synaptic polarities out of the space of 738 unresolved polarities, altogether identifying 24 polarities at 95% precision, as illustrated in Figure 2E. This is much better than a similar method based on paths of length two, that is, the principle of structural balance,^[^ ^26^ ^]^ signed length 2 (SL2) (see Section 4), identifying only one polarity at 95% precision (**Figure** **5**).

Yet, SL3 is just one of the many existing and emerging network‐based sign prediction methods. For example, another simple yet successful approach is that based on preferential attachment (PA).^[^ ^27^ ^]^ In the simplest implementation of PA, the probability of a connection is assumed to be proportional to both node degrees. Here, we extend PA to the signed, directed case by representing positive and negative edges as separate network layers and using the difference in their PA predictions to make signed predictions (signed preferential attachment [SPA] in Section 4). SPA identifies 49 complex polarities at 95% precision, corresponding to an absolute prediction score of ⩾66. At the same threshold SPA unveils an additional 63 synaptic polarities, altogether identifying 112 polarities at 95% precision, as illustrated in Figure 2F. Our findings indicate that signed extensions of link prediction methods,^[^ ^28^ ^]^ as well as the growing body of network sign prediction methods^[^ ^29^, ^30^, ^31^, ^32^ ^]^ should be further explored.

## 3 Conclusions

Understanding the structure and function of the brain remains one of the most elusive goals across all scientific disciplines. The synaptic connectome, which comprises all excitatory and inhibitory synaptic connections between neurons, plays a major role in encoding the possible functions that the brain can achieve. With an increasing amount and quality of input data on synaptic polarities in various organisms, both gene expression (CM/SCM) and signed network‐based (GCM, SL3, SPA) methods are expected to provide useful predictions for still missing synaptic polarities, considerably expanding the signed connectome. The ultimate goal is to provide sufficient inputs for models of neuronal dynamics to offer insights into how high‐level behaviors are encoded into neuronal circuits, especially in higher organisms.^[^ ^1^, ^2^, ^3^, ^33^ ^]^

While we introduced computational tools to address various experimental scenarios—even one without any genetic information—detailed NT and R expression profiles are highly desirable as they enable the use of the superior CM and SCM, compared to GCM or current network‐based methods. Remarkably, we have found that the SCM can achieve high precision even from a small fraction of the input data, in particular 5% of the known polarities is already sufficient to infer most polarities at 95% precision. This indicates that even a mostly incomplete subset of synaptic polarities might serve as a starting point for reliable predictions in higher organisms.

Our results are just the first steps toward a better understanding of synaptic polarity patterns in higher organisms, such as humans. In particular, when considering no gene expression or wiring rules, network sign prediction needs to be further explored. We note that the signed, directed generalization of L3, PA, and other network‐based link prediction methods can be done in several alternative ways and here we only scratch the surface of what could be achieved by such signed network‐based techniques. For example, appropriate degree‐normalization could significantly improve the performance of such network‐based methods, as well as including additional node features. As a fundamental limitation, computational approaches are restricted to infer polarities between nodes with some existing information. In other words, purely computational methodologies cannot add new nodes to the input network, but can make the information more dense in the sense of assigned synaptic polarities.

In lack of more suitable large‐scale datasets, we have illustrated our proposed methods on the example of the *C. elegans* synaptic connectome. Although this is a well studied model organism, our information on NT and R expression as well as the wiring rules and known synaptic polarities is still limited. Moreover, most “known” synaptic polarities are still awaiting experimental confirmation. Due to these factors and the relatively small size of the *C. elegans* connectome, we must be careful when interpreting the results. To improve our methods further, it will be essential to obtain large‐scale polarity maps, where the polarity of each synapse is experimentally confirmed. Without such datasets, we are limited to draw conclusions based on computational validations. Here, we chose to accept the CM‐resolved complex polarities as a ground truth. As an alternative, we also considered traditional *k*‐fold cross validation (see Section 4), leading to even higher precision values (Figure 3E). While the SCM provided next to perfect precision in this cross validation, both SL3 and SPA performed similarly well. However, such a traditional analysis might overestimate the real life performance of the methods, as the input datapoints are neither independent nor balanced. For example, erroneous wiring rules or gene expression could lead to correlated, large‐scale deviations, significantly impacting the results. Nevertheless, methods that do not perform comparably well in cross validation are unlikely to work well in real life scenarios either.

Finally, we mention that, assuming that the input datasets are of high quality, our proposed framework could also be used to computationally assess the significance of each predicted polarity. The idea^[^ ^23^ ^]^ is to consider a randomized network ensemble,^[^ ^34^ ^]^ where not only the network topology, but also the signed degree of each node is preserved—at least on average. In such a random ensemble, there are no wiring rules apart from the constraints posed by the signed degrees of individual nodes. As a result, the predicted polarities should correspond to a “random expectation” on average, a value that is typically non‐zero. The statistical significance of each predicted polarity in the real life data can then be assessed by a *z*‐score compared to that obtained for members of the randomized network ensemble. As the CM and the SCM are linear in the input polarities, a computationally efficient solution is feasible, by extending the randomization protocol introduced in the original formulation of the SCM.^[^ ^23^ ^]^ Alternatively, one could keep the network data intact while suitably randomizing the gene expression profiles. We leave these investigations for future studies, as they will gain more relevance with experimentally confirmed polarities as an input. In the meanwhile, to further support the robustness of our results, we have repeated our main calculations with alternative connectome reconstructions,^[^ ^8^, ^9^ ^]^ leading to qualitatively similar results, as illustrated in Figure 3F.

**Figure 6:**
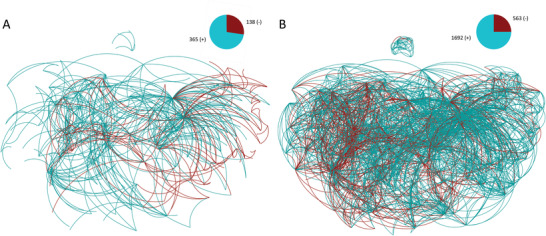
Summary of inferred polarities: A) The union of the 356 CM‐resolved complex and the rest of the 147 SCM‐resolved polarities above the selected 95% precision threshold. B) The union of our inferred polarities from panel (A) and the known network, resulting in 2255 resolved polarities.

To achieve the best overall predictions in the *C. elegans*, we recommend to extend the known network with the 356 complex connections resolved by the CM, in addition to the rest of the 147 predictions of the SCM at a strict 95% precision threshold **Figure** **6**A. Altogether, at the same threshold, the CM and SCM infers 138 inhibitory connections, a 32% increase compared to those in the known network. At the same time, we infer 365 excitatory connections, a 28% relative increase. As a sign of consistency, the E:I balance at the connection level has almost no shift (from 76:24 to 75:25), with the addition of 503 inferred polarities, as shown in Figure 6. At the level of synapses, both the known and the extended *C. elegans* synaptic connectome has an E:I balance of 78:22, regardless of the inclusion of the 3190 synapses corresponding to the 503 resolved polarities. In comparison, in humans, a cubic millimeter sample in volume has been recently made available,^[^ ^1^ ^]^ containing ≈50000 neurons and glia. The polarity of synapses was predicted by a classification model that considered the EM imagery centered around each putative synapse, as well as the local pre‐ and postsynaptic neuron segment masks. Within this dataset the E:I balance of synapses was found to be 63:37.

An interesting feature of the SCM is the construction of an abstract generalized wiring rule network, minimal in the sense of squared weights. Although such a network is apparently a useful intermediate step, there is no guarantee that it is directly relevant biologically. While it is plausible to assume that the true biological wiring rules are nearly optimal also in some biological sense, it is not clear if it is captured by the simple prescription of least square rule weights. In addition, systematic errors in gene expression could potentially lead to auxiliary compensatory wiring rules in the model. Yet, considering the high predictive power of the SCM, it is an intriguing possibility to experimentally study some of the novel SCM wiring rules in Figure 4A, in addition to the inferred polarities.

## 4 Experimental Section

### Inferring New Polarities with the SCM

The SCM^[^ ^23^ ^]^ was used to infer a minimal set of connection rules $\tilde{O}$, with the signed connectome, A, at hand. In the SCM of the studied 295 neurons, the known polarities *A*_*ij*_, wiring rules ${\overset{}{O}}_{\mathrm{ij}}$, and the synaptic connectome *C*_*ij*_ were represented as vectors, that is, *a*_295*i* + *j*_ = *A*_*ij*_, ${\overset{}{o}}_{295i+j}={\overset{}{O}}_{\mathrm{ij}}$, and *c*_295*i* + *j*_ = *C*_*ij*_ respectively, where *a*_295*i* + *j*_ ≠ 0 in the space of connections with known polarities only, of *c*_295*i* + *j*_ = 1. Entries corresponding to *c*_295*i* + *j*_ = 0 and entries labeled as "complex" were truncated out of *a*_295*i* + *j*_ as well as the corresponding rows of K, yielding $a^{\prime }$ and $K^{\prime }$, respectively. Then, $\tilde{o}$ was found using ridge regression

(6) $$\underset{\tilde{o}}{\arg\;\min}\left|\right|K^{\prime }\tilde{o}-a^{\prime }{\left|\right|}^{2}+\alpha ||\tilde{o}{\left|\right|}^{2}$$

where the regularization parameter α was inroduced that controls the magnitude of the NT‐R rules. Optimizing Equation (6) with respect to $\tilde{o}$ leads to

(7) $$\tilde{o}={\left(K^{\prime ⊺}K^{\prime }+\alpha I\right)}^{-1}K^{\prime ⊺}a^{\prime }$$

According to Equation (7), in the heavily regularized limit of α → ∞, $\tilde{o}\propto K^{\prime ⊺}a^{\prime }$, while in the opposite limit of α → 0, $\tilde{o}=K^{\prime †}a^{\prime }$, where $K^{\prime †}$ is the Moore–Penrose pseudoinverse of $K^{\prime }$. The optimal value of the hyperparameter α was chosen to maximize the rank at which 95% precision was achieved.

### Signed Paths of Length Two

As a traditional starting point, the triadic closure principle (TCP) postulates a higher connection probability between two nodes that share a large number of neighbors,^[^ ^35^ ^]^ i.e. a higher number of paths connecting them at length two. In an undirected, unsigned network the simplest manifestation of TCP is known as the common neighbors (CN) method, prioritizing predictions as entries in the similarity matrix $S=A^{2}$, where A stands for the adjacency matrix. The same formula could be applied to undirected, signed inputs as well, capturing structural balance at the triangle level,^[^ ^36^ ^]^ summarizing that “a friend of my friend is my friend”, “a friend of my enemy is my enemy” and “an enemy of my enemy is my friend”. However, there are multiple possible generalizations to a directed network with an asymmetric A matrix, such as $S_{\mathrm{in}}=A^{⊺}A$ or $S_{\mathrm{out}}={\mathrm{AA}}^{⊺}$, etc. While $S_{\mathrm{in}}$ counts the number of shared presynaptic neurons, $S_{\mathrm{out}}$ counts the postsynaptic neurons. Based on the observed performance in computational cross validation, $P_{\mathrm{SL}2}=S_{\mathrm{in}}+S_{\mathrm{out}}$ was chosen as the representative TCP method.

### Signed Paths of Length Three

As observed in ref. [25], triangles (such as TCP) might have limited predictive power in biological networks, while considering longer paths could lead to significant improvements, especially paths of length 3 (L3). A natural starting point for an alternative methodology structured around paths of length 3 is $A^{3}$. A signed directed generalization of L3 is proposed as

(8) $$P_{\mathrm{SL}3}={\mathrm{AA}}^{⊺}A\;$$

counting paths of length three that made the first and last steps forward while one step back along the paths as indicated in Figure 5. Such a choice was motivated simply by the matching dimensions of the multiplied matrices. In addition, the SL3 formula has a direct intuitive interpretation. For example, in SL3, a post‐synaptic partner is inhibited if the majority of similar partners were inhibited by the presynaptic neuron, $P_{\mathrm{SL}3}\equiv {\mathrm{AS}}_{\mathrm{in}}$, where similarity was assessed based on incoming connections. Simultaneously, a pre‐synaptic partner is inhibitory if similar presynaptic neurons were mostly inhibitory, $P_{\mathrm{SL}3}\equiv S_{\mathrm{out}}A$, where similarity was assessed based on outgoing connections.

### Signed Preferential Attachment

As an alternative to path‐based methods, the simplest manifestation of preferential attachment states that the connection probability between two nodes is proportional to their degrees, that is their number of neighbors, *k*_*i*_ and *k*_*j*_, leading to *p*_*ij*_ ≈ *k*_*i*_ *k*_*j*_. Our—arguably simplest— generalization to signed, directed networks is $p_{ij}=k_{i}^{+,\mathrm{out}}k_{j}^{+,\mathrm{in}}-k_{i}^{-,\mathrm{out}}k_{j}^{-,\mathrm{in}}$. Intuitively, this choice models the signed network as the sum of a positive and negative network, each driven independently by preferential attachment.

### Computational Cross Validation

As a traditional measure of the effectiveness of the results, a *k*‐fold cross validation procedure was implemented. *k*‐fold cross validation entails segmenting the space of connections with known polarity into *k* equal and randomly chosen “folds.” Each method was “trained” on each combination of *k* − 1 folds as an input and “tested” by comparing the trained methods predictions to the known sign of each edge in the remaining “test” fold to estimate how accurately each representative method generalized to unseen data. The predictions were ordered by their magnitude and assigned $a_{i}=\delta_{\tilde{s}\left(i\right),s\left(i\right)}$, where *s*(*i*) ($\tilde{s}\left(i\right)$) were the known (predicted) polarities at rank *i*, and δ is the Kronecker delta which is one when the signs match and zero otherwise. The precision at rank *r* for the *k*th fold was then given as $p^{\left(k\right)}\left(r\right)=\frac{1}{r}\sum_{i=1}^{r}a_{i}^{\left(k\right)}$. The mean of the precision over *k* folds was plotted for each method. Note that in the case of the SCM, the input data for cross validation was truncated to remove the test fold.

### Network Analyses

Network visualization had been obtained by the EntOpt plug‐in of Cytoscape version 3.7.2.^[^ ^37^, ^38^ ^]^

## Conflict of Interest

The authors declare no conflict of interest.

## Author Contributions

The manuscript was written with contributions from all authors. The work was conceived and supervised by I.A.K. M.R.H. implemented the main prediction methods. T.P.W. optimized the regularization hyperparameters. I.A.K. developed the models, analyzed, and interpreted the numerical results.

## Data Availability

The data that support the findings of this study are openly available in zenodo at https://doi.org/10.5281/zenodo.6342306, reference number 6342306.
